# Sini San Inhibits Chronic Psychological Stress-Induced Breast Cancer Stemness by Suppressing Cortisol-Mediated GRP78 Activation

**DOI:** 10.3389/fphar.2021.714163

**Published:** 2021-11-29

**Authors:** Yifeng Zheng, Juping Zhang, Wanqing Huang, Linda L. D. Zhong, Neng Wang, Shengqi Wang, Bowen Yang, Xuan Wang, Bo Pan, Honglin Situ, Yi Lin, Xiaoyan Liu, Yafei Shi, Zhiyu Wang

**Affiliations:** ^1^ The Research Center of Integrative Cancer Medicine, Discipline of Integrated Chinese and Western Medicine, The Second Clinical College of Guangzhou University of Chinese Medicine, Guangzhou, China; ^2^ State Key Laboratory of Dampness Syndrome of Chinese Medicine, The Second Affiliated Hospital of Guangzhou University of Chinese Medicine, Guangzhou, China; ^3^ Guangdong-Hong Kong-Macau Joint Lab on Chinese Medicine and Immune Disease Research, Guangzhou University of Chinese Medicine, Guangzhou, China; ^4^ School of Chinese Medicine, Hong Kong Baptist University, Kowloon, China; ^5^ The Research Center for Integrative Medicine, School of Basic Medical Sciences, Guangzhou University of Chinese Medicine, Guangzhou, China; ^6^ Guangdong Provincial Key Laboratory of Clinical Research on Traditional Chinese Medicine Syndrome, Guangdong Provincial Academy of Chinese Medical Sciences, Guangdong Provincial Hospital of Chinese Medicine, Guangzhou, China

**Keywords:** Sini San, breast cancer stem cells, chronic psychological stress, GRP78/LRP5, cortisol

## Abstract

Chronic psychological stress is closely correlated with breast cancer growth and metastasis. Sini San (SNS) formula is a classical prescription for relieving depression-related symptoms in traditional Chinese medicine (TCM). Current researches have suggested that chronic psychological stress is closely correlated with cancer stem cells (CSCs) and endoplasmic reticulum (ER) stress. This study aimed to investigate the effects of chronic psychological stress on ER stress-mediated breast cancer stemness and the therapeutic implication of SNS. Chronic psychological stress promoted lung metastasis in 4T1 breast tumor-bearing mice and increased the stem cell-like populations and stemness-related gene expression. Meanwhile, GRP78, a marker of ER stress, was significantly increased in the breast tumors and lung metastases under chronic psychological stress. As a biochemical hallmark of chronic psychological stress, cortisol dramatically enhanced the stem cell-like populations and mammospheres formation by activating GRP78 transcriptionally. However, GRP78 inhibitors or shRNA attenuated the stemness enhancement mediated by cortisol. Similarly, SNS inhibited chronic psychological stress-induced lung metastasis and stemness of breast cancer cells, as well as reversed cortisol-induced stem cell-like populations and mammospheres formation by attenuating GRP78 expression. Co-localization and co-immunoprecipitation experiments showed that SNS interrupted the interaction between GRP78 and LRP5 on the cell surface, thus inhibiting the Wnt/β-catenin signaling of breast CSCs. Altogether, this study not only uncovers the biological influence and molecular mechanism of chronic psychological stress on breast CSCs but also highlights SNS as a promising strategy for relieving GRP78-induced breast cancer stemness *via* inhibiting GRP78 activation.

## Introduction

Long-term and frequent repeated exposure to psychological stress has been reported to influence the normal functions of various organs (Hering et al., 2015). Chronic psychological stress is increasingly considered a risk factor in the development and progression of cancer (Krizanova et al., 2016). Strong involvement and impact of chronic psychological stress have been found in multiple malignancies (Batty et al., 2017). According to a previous survey, the prevalence of any mental health disorder was highest among breast cancer patients (41.6%; 95% CI, 36.8–46.4%) (Mehnert et al., 2014). Our previous meta-analysis of 282,203 breast cancer patients indicated that depression/anxiety was an independent factor in predicting breast cancer recurrence and survival (Wang et al., 2020). Therefore, it is necessary to develop complementary treatment strategies to improve the outcomes of breast patients with chronic psychological stress.

CSCs are recognized as the origin of cancer progression. Metastasis, drug resistance, and cancer relapse were reported to rely on the maintenance of CSCs stemness (Nassar and Blanpain, 2016). A recent study has indicated the potential role of chronic psychological stress on cancer stemness. Chronic psychological stress stimulated lactate production in breast cancer by elevating LDHA. The decreased pH caused by LDHA generating lactate mediated the stabilization of Myc, thereby promoting breast cancer stem-like properties (Cui et al., 2019). Moreover, CSCs express relatively higher levels of stress-related genes than other cancer cells. Thus, environmental stress could upregulate the expression of stress proteins and enhance cancer stemness (Tower, 2012). ER stress plays a crucial role in regulating CSCs in the stress signaling network. The inhibition of ER stress receptors was shown to promote the apoptosis of CSCs (Tower, 2012). Notably, GRP78 is an essential molecular marker of ER stress. The self-renewal and tumorigenic ability of head and neck CSCs were attenuated if the cell membrane expression of GRP78 was blocked (Lee et al., 2014). Interestingly, chronic psychological stress not only influences neuroendocrine function but also activates ER stress in response to external stress. Previous studies have proven that chronic psychological stress causes ER stress activation in the brain and susceptible tissues (Chad-Friedman et al., 2017; Antoni and Dhabhar, 2019; Wang et al., 2019). Therefore, ER stress and its molecular marker GRP78 might be the key to block the relationship between cancer stemness and chronic psychological stress.

Elevation of plasma glucocorticoid levels is one of the characteristics induced by chronic psychological stress (Cui et al., 2020). Related studies have suggested that elevated glucocorticoids in cancer patients mediate the release of inflammatory factors and tumor immunosurveillance, promoting tumor heterogeneity and metastasis (Cui et al., 2020). Notably, in the last 2 years, some studies have found that glucocorticoids (GCs) and glucocorticoid receptors (GRs) play a crucial role in CSCs regulation. GR antagonism inhibited breast CSCs formation by reducing the expression of YAP protein (Kim et al., 2020). In addition, GCs activated the interaction between GR and domain transcription factor 4 (TEAD4), promoting breast CSCs maintenance, cell survival, metastasis, and chemotherapy resistance (He et al., 2019). Therefore, the excessive glucocorticoids induced by chronic psychological might be a starting point of the CSCs signaling network.

SNS is composed of *Bupleurum chinense* DC. (*Radix Bupleuri*), *Paeonia lactiflora* Pall. (*Radix Paeoniae Alba*), *Citrus × aurantium* L. (*Fructus Aurantii Immaturus*), and *Glycyrrhiza uralensis* Fisch. (*Radix Glycyrrhizae*). It has been used to relieve emotional disorders in TCM for a long time. Current clinical research suggested that the combination of SNS and paroxetine could significantly increase the level of 5-hydroxytryptamine and reduce cortisol, which conferred promising efficacy in patients with depression (Wu et al., 2019). Interestingly, receiving appropriate treatment for chronic psychological stress may also improve the prognosis of cancer patients (Ferrell et al., 2015; Zhang et al., 2020). Traditional Chinese medicine (TCM) has become an important part of adjuvant oncological treatment. Some TCM prescriptions could relieve the chronic stress induced by cancer pain and reduce psychological distress caused by cancer diagnosis and treatment, thus restricting cancer progression (Liu et al., 2017; Sun et al., 2018; Dang et al., 2019). A meta-analysis showed that various TCM prescriptions, including SNS, could alleviate the depressive symptoms of cancer patients (Li et al., 2019). Notably, it has been reported that SNS reduces the risk of recurrence and metastasis of estrogen and progesterone receptor-negative breast cancer in the clinical practice of TCM (Gao, 2019). However, the underlying molecular mechanisms by which SNS suppresses breast cancer progression induced by chronic stress have not been established.

In this study, we confirmed that chronic psychological stress could promote breast cancer stemness in a GC-dependent manner by activating GRP78. More importantly, SNS inhibited breast cancer stemness by interrupting the interaction between GRP78 and LRP5 induced by chronic psychological stress. Our study not only uncovers the potential link between chronic psychological stress and GRP78 activation in breast CSCs, but also highlights SNS as a potential therapy to improve the stress condition and clinical outcomes of breast cancer patients.

## Materials and Methods

### Preparation of the SNS Formula

The SNS formula was prepared according to the clinical prescription. The herbs including 10 g *Bupleurum chinense* DC. (*Radix Bupleuri*) (2008002, Lingnan Traditional Chinese Medicine Tablets, Foshan, China), 10 g *Paeonia lactiflora* Pall. (*Radix Paeoniae Alba*) (200802, Zisun Chinese pharmaceutical, Guangzhou, China), 10 g *Citrus × aurantium* L. (*Fructus Aurantii Immaturus*) (200701, Zisun Chinese pharmaceutical, Guangzhou, China), 10 g *Glycyrrhiza uralensis* Fisch. (*Radix Glycyrrhizae*) (2007001, Lingnan Traditional Chinese Medicine Tablets, Foshan, China) were soaked for 1 h and refluxed at 95°C with an 8-fold volume of distilled water for 1 h. The extracts after filtration were condensed by rotary evaporation at 65°C and freeze-dried to obtain the lyophilized powder. The yield ratio of SNS was 21.96% (w/w). According to the clinical prescription and the best practice statement in phytopharmacological research, SNS was administrated to mice at 0.825 and 1.65 g/kg every day, respectively (Heinrich et al., 2020). The concentration gradients set for the *in vitro* experiments were according to the preliminary experimental results. The fingerprint of SNS was determined by the high-performance liquid chromatography (HPLC) analysis. For the quality of SNS, four major compounds (saikosaponin A, naringin, paeoniflorin, glycyrrhizic acid) were used as quality control standards, and the contents of the standard compounds were also measured.

### Cell Culture

4T1 murine breast cancer cell line was purchased from American Type Culture Collection (ATCC) and cultured in Dulbecco’s Modified Eagle Medium (DMEM, Gibco, NY, United States), supplemented with 10% fetal bovine serum (Gibco, NY, United States) and 1% penicillin and streptomycin (Gibco, NY, United States) at 37 °C in a humidified incubator containing 5% CO_2_. The identity had been authenticated by short tandem repeat profiling and tested for mycoplasma contamination.

### Western Blot

The protein lysates were applied to SDS-PAGE, transferred to a PVDF membrane (Millipore, Billerica, MA, United States) and probed with primary antibodies and amplified by the secondary antibodies. The primary antibodies included GRP78 antibody (11587-1-AP, Proteintech, Rosemont, IL, United States), β-catenin antibody (51067-2-AP, Proteintech, Rosemont, IL, United States), LRP5 (24899-1-AP, Proteintech, Rosemont, IL, United States), β-actin antibody (4,970, Cell Signaling Technology, Danvers, MA, United States), *p*-LRP5 (AF4345, Affinity Biosciences, Cincinnati, OH, United States). Finally, the bands were imaged through the ECL Advance reagent (Tanon Science and Technology, Shanghai, China) and quantified by optical densities using the ImageLab software (BIO-RAD, Hercules, CA).

### qPCR

Total RNA was extracted with RNAiso Plus Reagent (Takara BIO, Shiga, Japan) and reverse transcribed to complementary cDNA using the PrimeScript™ RT reagent Kit (Takara, Shiga, Japan) following the manufacturer’s instructions. Briefly, total RNA was extracted and then evaluated by measuring the A260/A280 ratio. Complementary DNA was generated from 1 g total RNA by reverse transcription after gDNA erasing. RT-PCR was performed using the SYBR Premix Ex Taq Kit II kit (Takara BIO, Shiga, Japan) and Biosystems QuantStudio 7 Flex Real-Time PCR System (Thermo Fisher Scientific, Hudson, United States). PCR amplicons were detected by fluorescent detection of SYBR green. The relative mRNA levels were compared using the 2-^ΔΔCt^ method.

### Transfection of Plasmids

The commercialized recombinant plasmids and shRNA plasmid for GRP78 were purchased from Vigene Biosciences (Jinan, China). According to the manufacturer’s protocol, all the above plasmids were transfected into indicated cells using lipofectamine 3000 (Invitrogen, Carlsbad, CA, United States). Briefly, 10 μl of transfection reagent was mixed with 2.5 μg plasmid and added into cells to maintain 16 h. GRP78 expression of cells was verified by western blot.

### CSCs Population Analysis and Mammosphere Formation Assay

Hyperactive aldehyde dehydrogenase (ALDH) activity is closely related to the physiological properties of stem cells (Wang and Lei, 2017). In the present study, the ALDH staining assay was conducted to discriminate breast cancer stem-like cells. ALDH^+^ population analysis was conducted by flow cytometry using the ALDEFLUOR Stem Cell Identification Kit (no.01700, STEMCELL Technologies, Vancouver, Canada). Briefly, breast cancer cells were incubated with ALDEFLUOR at 37°C for 30 min to mark ALDH^+^ cells. After incubation, cells were washed with PBS and subjected to analysis using a flow cytometer (NovoCyte Quanteon 4,025 and NovoSampler Q System Bundle, Agilent Technologies, Shanghai and Beijing, China). DEAB (denotes diethylaminobenzaldehyde), a specific inhibitor of ALDH activity, is used to control for background fluorescence in the ALDH staining assay. For mammosphere formation assay, 4T1 cells were cultured in ultralow attachment plates in DMEM/F12 medium supplemented with 1% penicillin-streptomycin (Gibco, NY, United States), 2% B27 supplement (Gibco, NY, United States), 20 ng/ml EGF, 5 μg/ml insulin and 0.4% bovine serum albumin (Sigma-Aldrich, Shanghai, China). The number and size of mammospheres were quantified microscopically.

### Co-Immunoprecipitation Analysis

Co-immunoprecipitation assay was carried out by the Pierce Co-Immunoprecipitation Kit (Thermo Fisher Scientific, Hudson, NH, United States) according to the manufacturer’s instructions. In brief, the GRP78 antibody was immobilized with resin. Next, 4T1 cells were lysed and added to the control agarose resin to avoid nonspecific interactions with the resin matrix. Then the immobilized resin was incubated with the 4T1 cell lysates to detect target protein LRP5 by immunoblotting. The quenched antibody coupling resin was a negative control.

### Immunofluorescence

For immunofluorescence analysis, cells or tissue specimens were fixed with 4% paraformaldehyde for 20 min, washed three times with PBS. In particular, to investigate the cellular co-localization of GRP78 and LRP5, the cells were permeabilized by 0.25% Triton X-100 in PBS for 20 min. To observe cell surface-localized GRP78 under different treatments, the cells or tissue specimens were labeled by 1 μM Dil without permeabilization. After blocking in 5% bovine serum albumin (Sigma-Aldrich, Shanghai, China) at room temperature for 30 min, cells or tissue specimens were incubated with primary antibody overnight at 4°C, followed by incubation with the fluorescence-conjugated secondary antibody for 1 h at room temperature in the dark. The primary antibody included GRP78 antibody (11587-1-AP, Proteintech, Rosemont, IL, United States), LRP5 (24899-1-AP, Proteintech, Rosemont, IL, United States); Fluorescently labeled anti-rabbit Alexa Fluor 488 and Alexa Fluor 555 conjugated antibodies were used as secondary antibodies. 4′, 6-diamidino-2-phenylindole (DAPI, Sigma) was used to visualize the nuclei. Dil (Thermo Fisher Scientific, Hudson, NH, United States) was used to label cell membrane according to the manufacturer’s protocol at 37°C for 30 min. Fluorescence images were obtained using an LSM710 confocal microscope (Zeiss, Jena, Germany).

### Mice Procedures

Five-week-old female Balb/c mice were obtained from the Beijing Vital River Laboratory Animal Technology Co., Ltd. Experimental treatments of all mice were reviewed and approved by the Institutional Animal Care and Use Committee of Guangdong Provincial Hospital of Chinese Medicine (ethics approval number: 2021075). Animal studies were carried out in line with the internationally accepted principles for laboratory animal use and care. The mice were fed in the specific pathogen-free ventilation chambers under an ambient temperature of 20–25°C and 45–50% relative humidity and given sterilized food and water. Chronic unpredictable mild stress (CUMS) of the rodent model is widely accepted to most closely mimic the chronic psychological stress of humans. The CUMS procedure was conducted following the previously published protocol (Willner, 1997) with minor modifications. In brief, chronic mild stress includes food and water deprivation for 24 h; cage tilted at a 45° angle for 24 h; restraint stress for 4 h; lighting at night for 12 h; clamping of the tail for 15 min; forced swimming in cold water (4–8°C) for 5 min, and soiled cages. The above stressors were randomly administered with 2-3 kinds every day. Mice were randomly divided into three different groups: control (n = 8), CUMS (n = 8), and CUMS + SNS (n = 8). One week after stress exposure, the orthotopic mouse model of breast cancer metastasis was established. Briefly, luciferase gene-tagged 4T1 cells were injected into mammary fat pads of each mouse (at the cell density of 5×10^6^ in 200 μl phosphate-buffered saline). Simultaneously, SNS (1.65 g/kg, according to the conversion of human clinical dosage) was administered *via* oral gavage every day. Tumor volume was measured every 2 days and calculated with the formula ([width] ^2^ × [length]/2). After 4 weeks of CUMS, behavioral changes were examined, including the open field test and the sucrose preference test. At the end of treatment, the mice were anesthetized by isoflurane inhalation and injected intraperitoneally with D-luciferin (PerkinElmer, Boston, United States) at 150 mg/kg for luminescent imaging. The bioluminescence of the lung colonization was imaged and quantified with the IVIS-Spectrum system (PerkinElmer, Boston, United States).

### Open Field Test (OFT) and Sucrose Preference Test (SPT)

The chronic psychological stress of mice was assessed through the open field test (OFT) and sucrose preference test (SPT). For OFT, the mice were individually placed at the center of an open-field arena for 6 min. The open-field arena is 40 × 40 × 40 cm (length × width × height). Panlab Smart3.0 (Harvard Apparatus, Shanghai, China) automatically tracked and recorded the locomotor activity of mice, and the total movement distance and immobility time were analyzed. For SPT, the mice were acclimated to two bottles of 1% sucrose solution the day before the experiment. On the day of the experiment, each mouse was housed in an individual cage. A bottle of 1% sucrose solution and a bottle of water were provided, and each mouse was free to access. After 6 h, the consumption of 1% sucrose solution and water was recorded. Sucrose preference (%) = sucrose intake (ml)/[sucrose intake (ml) + water intake (ml)] × 100%.

### Enzyme-Linked Immune Assay (ELISA)

The concentration of corticosterone, cortisol, and cortisone in the serum of mice exposed to CUMS was detected by the ELISA kit (Cortisol, CEA462Ge; Corticosterone, CEA540Ge; Cortisone, CEA067Ge) following the manufacturer’s protocols (Cloud-Clone Corp. Katy, TX, United States). Absorbance was measured at 405 nm in a microplate reader (Synergy HTX Multi-Mode Reader, BioTek, VT, United States). The glucocorticoid levels were calculated by the fitting curve of the absorbance value obtained from a series concentration of standards.

### Immunohistochemistry, Hematoxylin-Eosin Staining, and TUNEL Analysis

Tissue samples were fixed with 4% paraformaldehyde for 24 h, followed by standard tissue processing and embedding. The immunohistochemical analysis was performed as described previously (Zheng et al., 2019). Briefly, Paraffin-embedded sample sections were cut at 4 μm and dried overnight at 37°C. The sections were then deparaffinized in xylene twice for 10 min each and rehydrated using a graded series of ethanol. Endogenous peroxidase was inactivated by incubating the sections in 3% hydrogen peroxide for 30 min at room temperature. Antigen retrieval was performed by heating the slides in the sodium-citrate buffer. The slides were then subjected to incubation with primary antibodies at 4°C overnight in a moist chamber. The DAB detection system (ZSGB-BIO, Bejing, China) was applied as chromogenic agents. Hematoxylin-eosin staining was conducted according to standard procedures. *In situ* apoptosis was evaluated by the detection of fragmented DNA via TUNEL analysis. Paraffin-embedded tumor sections were treated as previously (Jiao et al., 2019). In brief, the sections were deparaffinized and treated with proteinase K to strip proteins from the nuclei. Then they were incubated with terminal deoxynucleotidyl transferase at 37°C for 1 h. Streptavidin FITC was then added to the samples, followed by incubation in a humidified chamber for 30 min at 37°C. The fluorescence intensity was visualized at an excitation wavelength of 450–500 nm and an emission wavelength of 515–565 nm. The fluorescence intensity represents the degree of *in situ* apoptosis.

### Statistical Analysis

Data were presented as mean ± standard deviation (SD). Mice were randomly grouped, with eight animals for each group. For other experiments, displayed was one representative out of three independent experiments. All statistical analyses were performed using Statistical Product and Service Solutions (SPSS) 20.0 software (Abbott Laboratories, Chicago, United States). We used Student’s t-test analysis for two-group comparisons (two-sides). The one-way ANOVA and the Dunnett’s or Bonferroni post hoc test were performed to compare multiple groups. ANOVA for repeated measurement was performed towards repeated measures data. *p* < 0.05 was considered statistically significant.

## Results

### SNS Inhibits Breast Cancer Metastasis Induced by CUMS

**FIGURE 1:**
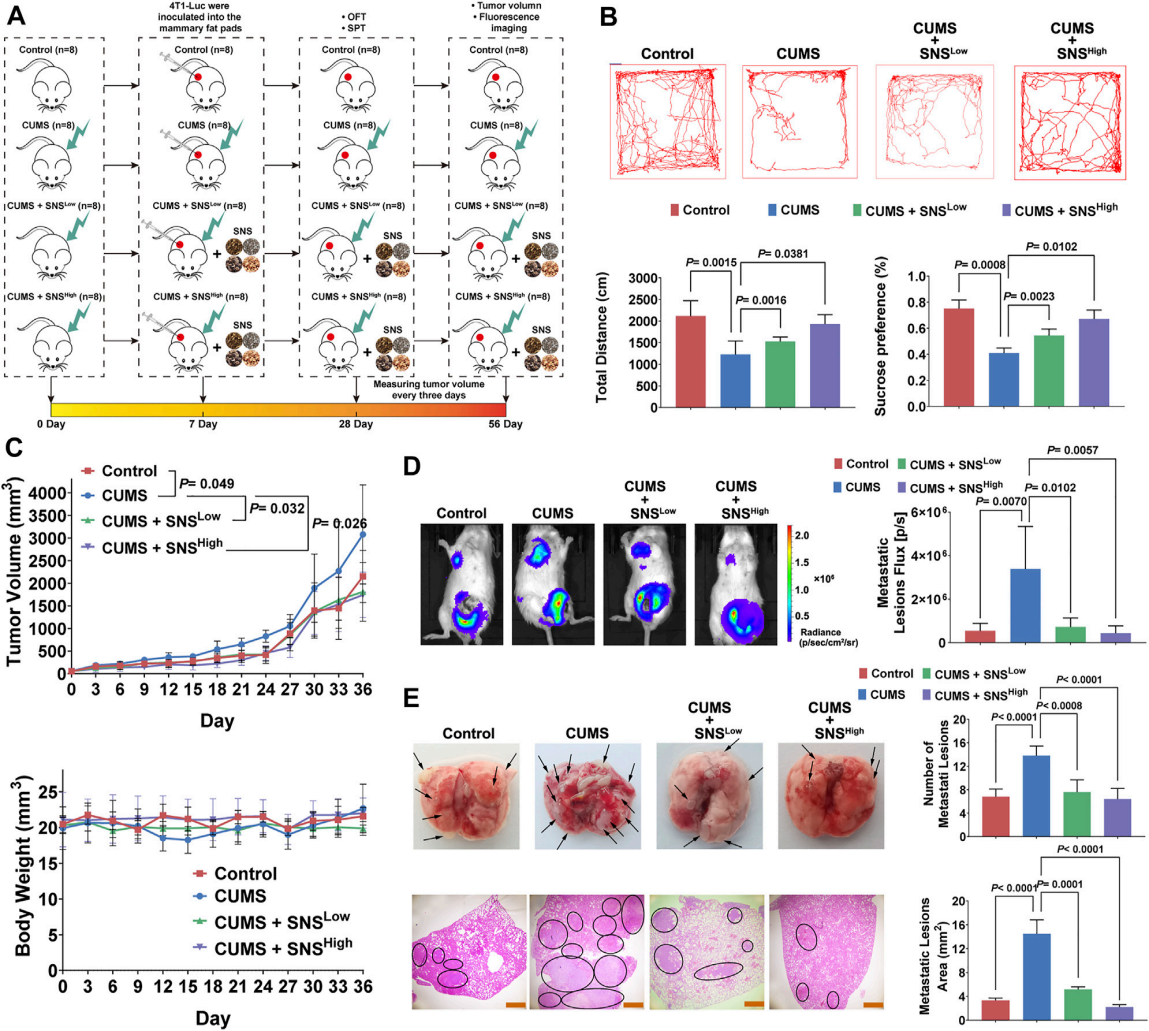
SNS inhibits CUMS-mediated lung metastasis of breast cancer in 4T1 tumor-bearing mice. **(A)** Flowchart of the experiment (SNS^Low^, 0.825 g/kg; SNS^High^,1.65 g/kg). **(B)** The representative movement trajectory of the mice (n = 8, upper panel), as well as the total distance, and percentage of sucrose water consumed (lower panel). **(C)** Tumor growth curves (upper panel) and body weight changes (lower panel) of the 4T1 tumor-bearing mice with different treatments (n = 8). **(D)** *In vivo* imaging of lung metastatic lesions in the 4T1 tumor-bearing mice with different treatments (left panel). The intensity of bioluminescence was quantified by an IVIS-spectrum system (n = 8, right panel). **(E)** Representative pictures of gross lung metastatic lesions in the 4T1 tumor-bearing mice with different treatments. The number of metastatic lesions was quantified (n = 8, upper panel). HE staining of the lung metastatic lesions in the 4T1 tumor-bearing mice with different treatments. The black circle indicates the metastatic lesions of the lung. The scale bars indicate 1000 μm. The metastatic lesion area was quantified by ImageJ software (n = 8, lower panel). Data are represented as the mean value ±SD. One-way ANOVA and Bonferroni’s post hoc test were applied. ANOVA for repeated measurements was performed in **(C)**.

CUMS in rodent models is widely accepted to most closely mimic the socio-environmental in human life, accounting for biochemical and behavioral abnormalities (Wu et al., 2015; Liu et al., 2019). Thus, we exposed orthotopic 4T1 tumor-bearing mice to CUMS to examine the effects of chronic psychological stress and SNS on breast cancer progression. Firstly, the fingerprint chromatogram and the contents of the standard compound of SNS were collected. The retention time of the selected standard compounds in the fingerprint chromatogram was consistent with that of the reference substance (Supplementary Figure 1). No significant differences were observed in three independent batches regarding the content of the standard compound (Supplementary Table 1). Thus, the quality of SNS was stable and controllable. The effects of SNS on tumor growth and metastasis under CUMS were measured as described in Figure 1A. The mice were exposed to CUMS for 7 consecutive days before inoculation with 4T1 cells *in situ*. The behavioral assays were performed on day 28 after the establishment of CUMS. SNS dose-dependently increased the total traveled distance in the open field test and daily sucrose water consumption (Figure 1B). Thus, SNS ameliorated the behavioral deficits induced by CUMS. Moreover, SNS inhibited the tumor growth induced by the CUMS stimulation (Figure 1C). Notably, CUMS facilitated lung metastases in mice with orthotopic breast cancer, as shown by *in vivo* imaging, but this phenomenon was reversed by SNS in a dose-dependent manner (Figure 1D). *Exo-vivo* observation of the lungs showed that SNS reduced the number of metastatic lesions. The HE staining results further indicated that SNS effectively reduced the metastatic lesion area (Figure 1E). Therefore, SNS could inhibit breast cancer growth and lung metastasis in mice stimulated by chronic psychological stress.

### CUMS Promotes the Stemness and GRP78 Expression of Breast Cancer Cells

**FIGURE 2:**
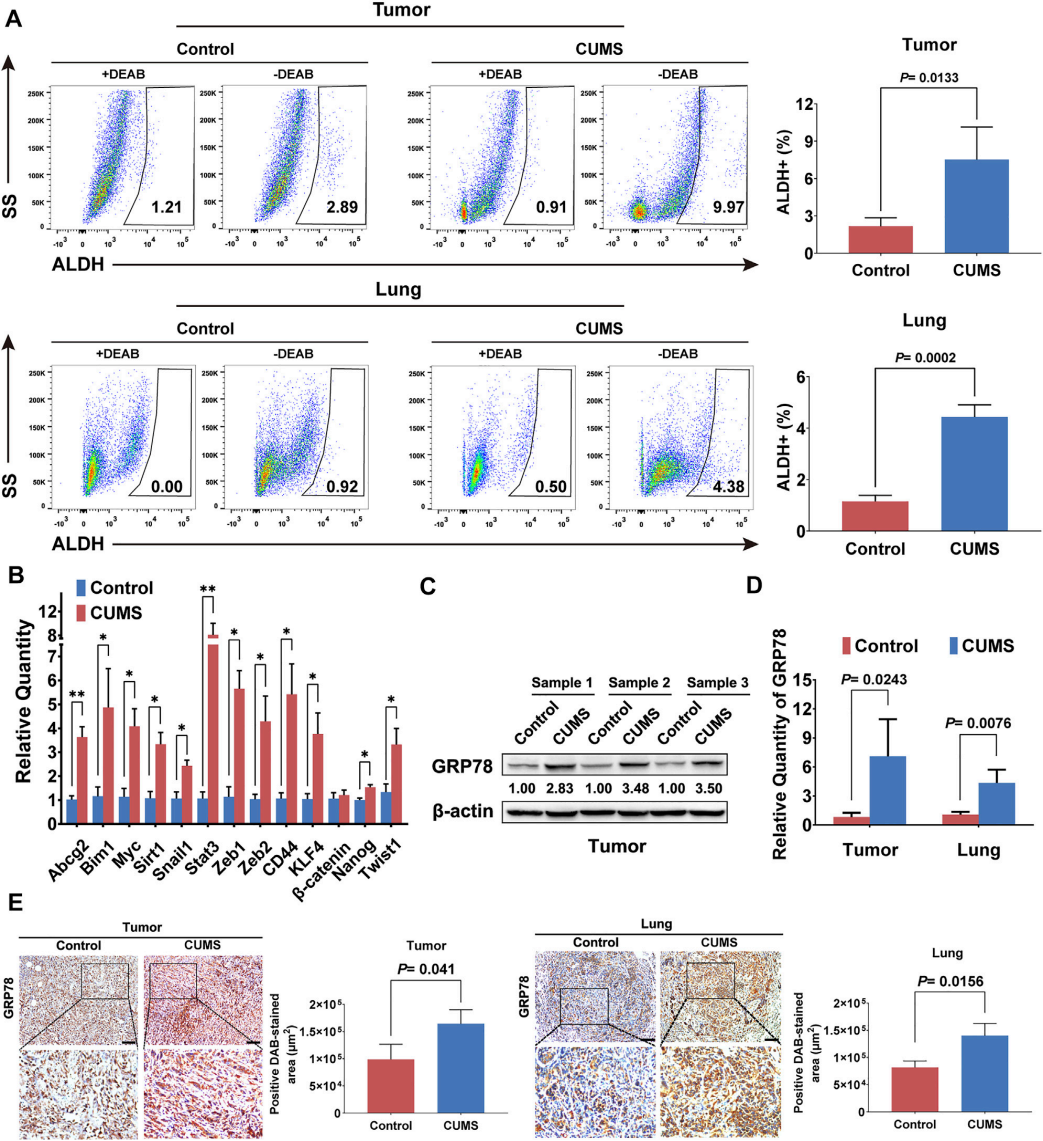
CUMS enhances GRP78 expression and breast cancer stemness in 4T1 tumor-bearing mice. **(A)** ALDH^+^ cells were analyzed by flow cytometry to compare the stem cell-like populations in primary tumor and lung metastatic lesions between the CUMS-treated and non-treated 4T1 tumor-bearing mice. DEAB, a specific inhibitor of ALDH activity, was used to control the background fluorescence. **(B)** Stem-related gene expression in the breast tumors under CUMS was compared with those in the non-treated mice by qPCR. **(C)** GRP78 expression in the primary tumors of the mice under CUMS was detected by western blots. **(D)** The mRNA level of GRP78 in the primary and metastatic lesions. **(E)** Expression of GRP78 in the primary and lung metastatic lesions in the CUMS and control groups was measured by immunohistochemistry (n = 8, the scale bars indicate 100 μm). Data are represented as the mean value ±SD. One representative experiment of three independent experiments is displayed. Unpaired two-sided Student’s t-tests were applied, *p < 0.05, **p < 0.01.

The stem cell-like properties of cancer cells are described as master gatekeepers and regulators of cancer growth and metastasis in multiple malignancies (Rich, 2016). The stem cell-like populations in primary breast cancer and lung metastases were significantly upregulated under CUMS, as shown by ALDH analysis (Figure 2A). The qRT-PCR results also indicated that most stemness-related genes were activated by CUMS (Figure 2B). Recent studies also reported that chronic psychological stress causes the activation of ER stress in the brain, liver, and other tissues, characterized by substantially increased expression of GRP78 (Mondal et al., 2015; Antoni and Dhabhar, 2019). Similarly, we found that CUMS also increased the expression of GRP78 in mouse breast tumor tissue. The transcript levels of GRP78 in the primary and lung metastatic lesions were also elevated (Figure 2C, D, Supplementary Figure 2A). Immunohistochemical staining of tumor and lung metastasis nodes further confirmed that the expression of GRP78 was significantly enhanced by CUMS (Figure 2E). These findings suggested that chronic psychological stress might enhance breast cancer stemness by elevating the ER stress protein GRP78.

### SNS Inhibits CUMS-Mediated Stemness and GRP78 Expression

**FIGURE 3:**
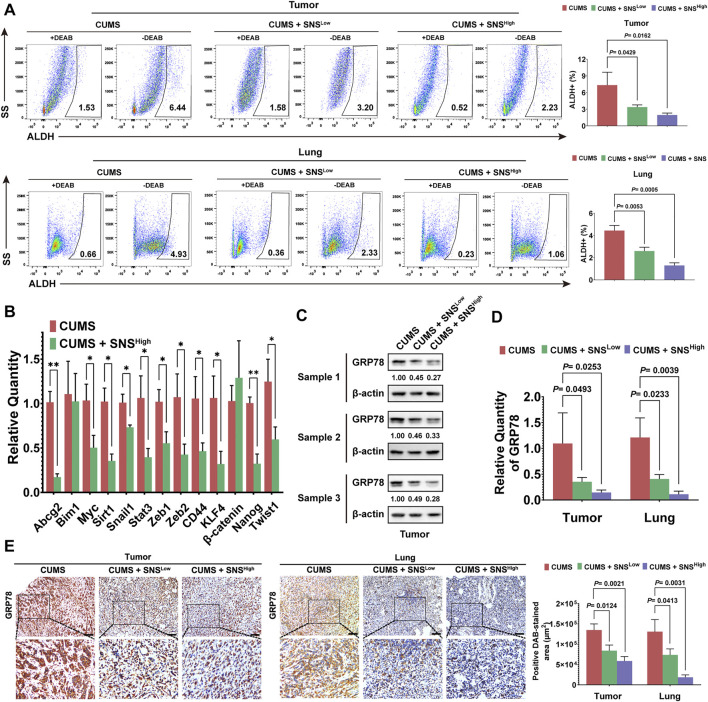
SNS inhibits CUMS-induced GRP78 expression and stemness of breast cancer cells in 4T1 tumor-bearing mice. **(A)** ALDH activity in the primary tumors and lung metastases were quantified after SNS treatment. **(B)** The relative quantity of stem-related genes in the primary tumor was detected by qPCR after SNS treatment. **(C)** The expression of GRP78 in the primary tumors was detected by western blots after SNS treatment. **(D)** Relative mRNA expression of GRP78 in the primary and lung metastatic lesions was measured. **(E)** Expression of GRP78 in the primary and lung metastatic lesions was detected by immunohistochemistry (n = 8, the scale bars indicate 100 μm). Data are represented as the mean value ±SD. One representative experiment of three independent experiments is displayed. Unpaired two-sided Student’s t-tests were applied, *p < 0.05, **p < 0.01.

We next investigated the effects of SNS on CUMS-induced stemness and GRP78 expression on breast cancer. SNS treatment was found to reduce the stem-like populations in primary tumors and lung metastases in a dose-dependent manner (Figure 3A). The qRT-PCR results also indicated that SNS downregulated the stemness-related genes activated by CUMS (Figure 3B). Subsequent detection showed that SNS inhibited GRP78 upregulation upon CUMS at both the protein and transcriptional levels (Figure 3C, D, Supplementary Figure 2B). Similarly, SNS significantly attenuated the expression of GRP78 in the primary tumors and lung metastasis of the CUMS-treated mice, as shown by immunohistochemistry (Figure 3E).

### Cortisol Elevated by CUMS Regulates the Stemness of Breast Cancer Cells *via* GRP78

**FIGURE 4:**
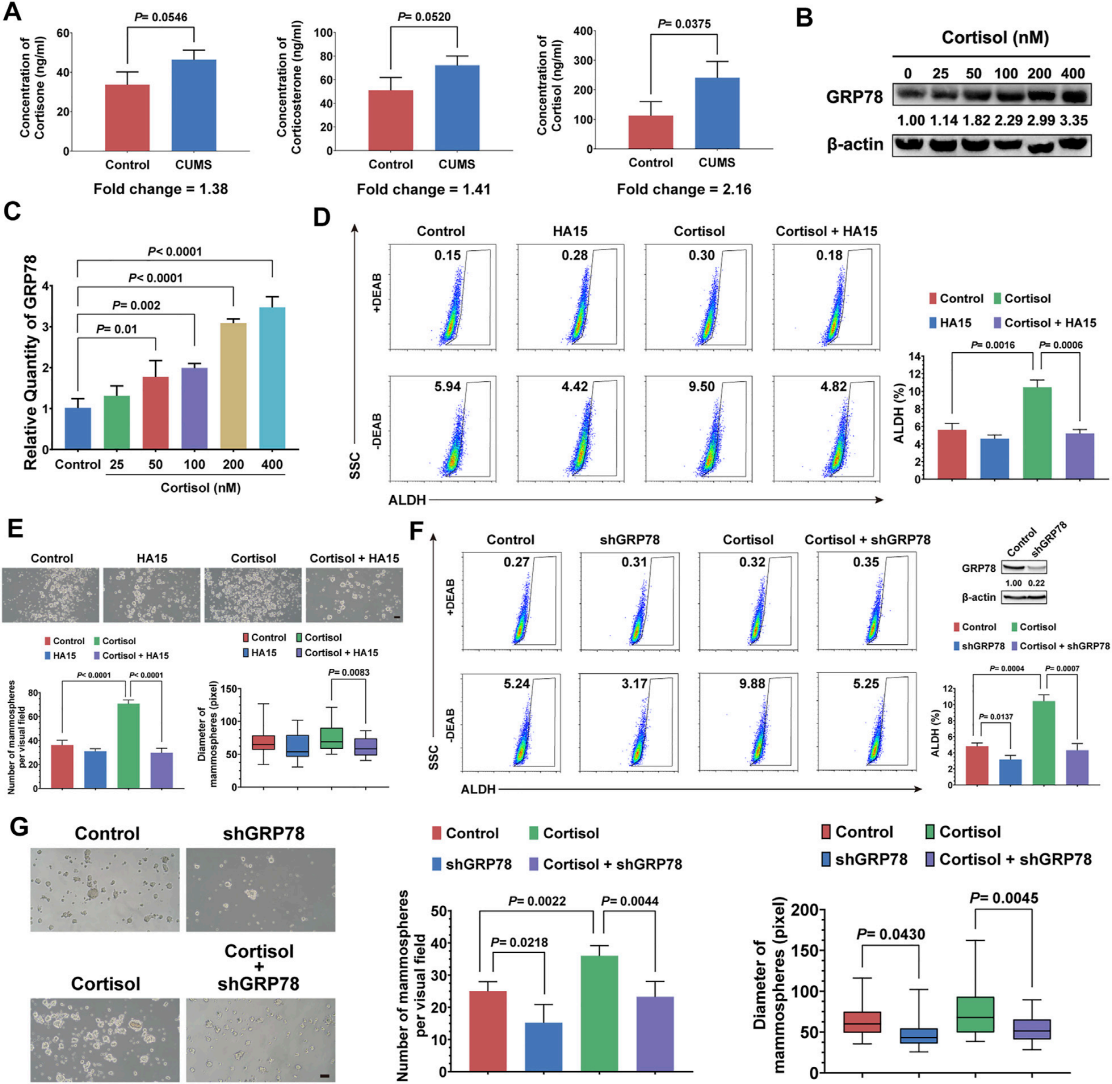
Cortisol elevated by CUMS enhances the stemness of breast cancer cells via GRP78. **(A)** The relative levels of cortisol, corticosterone, and cortisone in the plasma of the mice exposed to CUMS were measured by ELISAs. **(B)** GRP78 expression in 4T1 cells was detected by western blots after treatment with cortisol at a gradient concentration for 24 h. **(C)** The 4T1 cells were treated with cortisol at a gradient concentration for 24 h, and the relative mRNA expression of GRP78 was quantified by qPCR. **(D)** The ALDH^+^ cell population induced by cortisol was analyzed in the 4T1 cells after treatment with HA15 (10 μM), an inhibitor of GRP78, for 24 h. **(E)** The effect of HA15 on the size and number of the mammospheres induced by cortisol was quantified (the scale bars indicate 100 μm). **(F)** The ALDH^+^ cell population induced by cortisol was analyzed by flow cytometry following the GRP78 knockdown with shRNA (verification by western blot in the upper right panel). **(G)** The effects of GRP78 knockdown by shRNA on the size and number of the mammospheres induced by cortisol were examined (the scale bars indicate 100 μm). Data are represented as the mean value ±SD. One representative experiment of three independent experiments is displayed. Unpaired two-sided Student’s t-tests (A, D, E, F, G), one-way ANOVA, and Dunnett t-tests as post hoc tests (C) were applied.

CUMS mainly causes the activation of the hypothalamic-pituitary-adrenal (HPA) axis and sympathetic nervous system, both of which elevate circulating glucocorticoid levels (Zhang et al., 2020). Thus, we attempted to investigate the relationship between CUMS-induced high glucocorticoid levels and the stemness of breast cancer cells. First, we found that cortisol showed the most notable elevation in the breast tumor tissue induced by CUMS (Figure 4A). Moreover, the GRP78 expression of 4T1 cells was increased dose-dependent under cortisol treatment (Figure 4B, Supplementary Figure 3B). In accordance, cortisol also promoted the activation of GRP78 transcription (Figure 4C). Our previous studies revealed a close relationship between GRP78 and CSCs. Accordingly, cortisol increased the stem-like populations of 4T1 cells. However, HA15, a potent and specific inhibitor of GRP78, relieved the cortisol’s effects (Figure 4D). Similar results were found in the mammosphere formation assay, in which cortisol increased the size and number of the mammospheres while HA15 played the opposite effects (Figure 4E). GRP78 knockdown by shRNA inhibited the cortisol-induced stem-like populations and mammosphere formation capacity (Figure 4F, G, Supplementary Figure 3A). Therefore, excessive cortisol induced by chronic psychological stress enhanced the stemness of breast cancer cells, and GRP78 served as a critical adaptor molecule.

### SNS Inhibits the Stemness of Breast Cancer Cells by Suppressing GRP78

**FIGURE 5:**
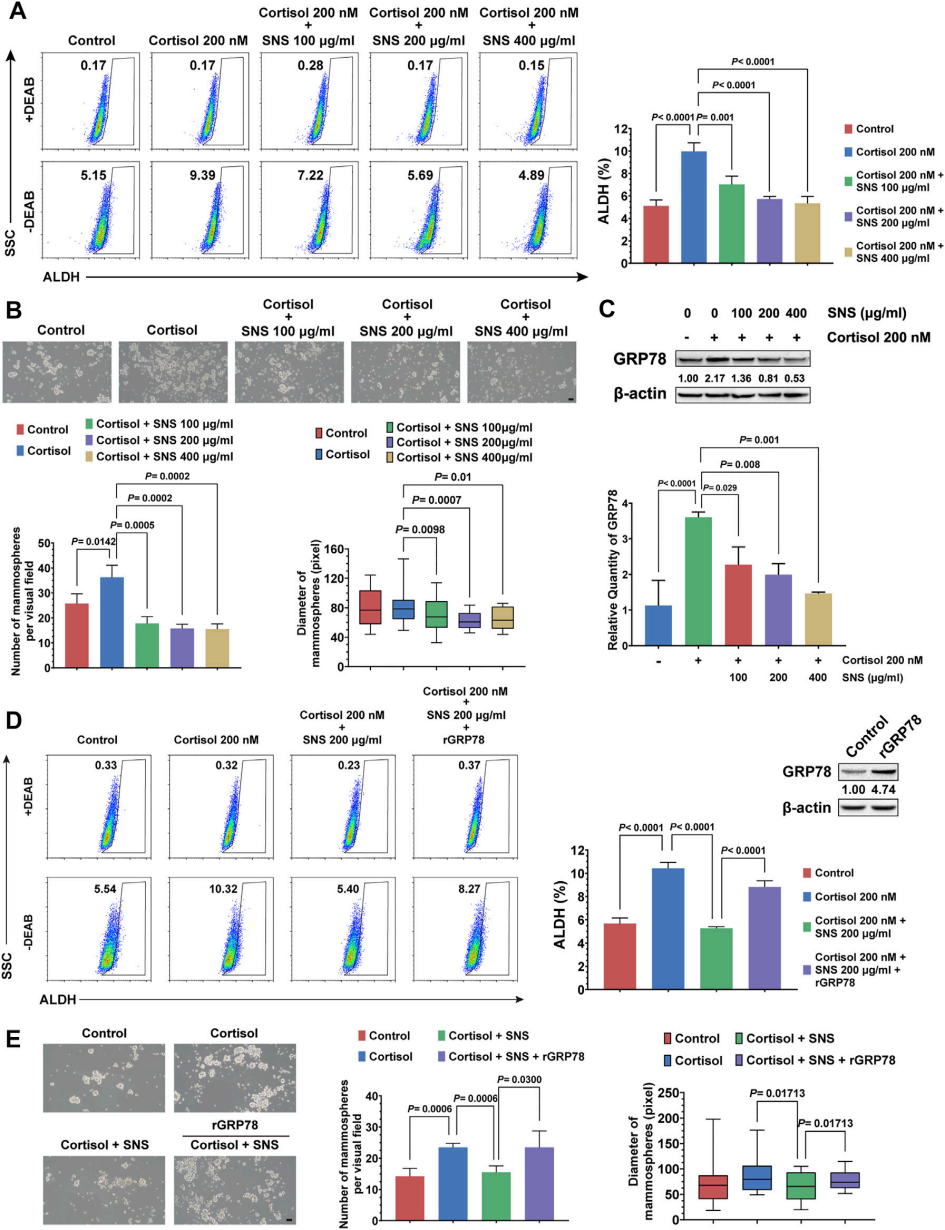
SNS inhibits the cortisol-induced stemness of breast cancer cells by suppressing GRP78. **(A)** The ALDH^+^ cell population induced by cortisol was analyzed in 4T1 cells after treatment with SNS at a gradient concentration for 24 h. **(B)** The size and number of mammospheres induced by cortisol were quantified following treatment with SNS for 24 h (the scale bars indicate 100 μm). **(C)** Following SNS treatment for 24 h, the protein and mRNA expression of GRP78 in the 4T1 cells under cortisol treatment was measured by western blots (upper panel) and qPCR (lower panel), respectively. **(D)** The effect of GRP78 overexpression on the anti-CSCs activity of SNS was analyzed by flow cytometry. **(E)** The effect of GRP78 overexpression on the inhibited activity of SNS on the mammosphere formation ability was analyzed (the scale bars indicate 100 μm). Data are represented as the mean value ±SD. One representative experiment of three independent experiments is displayed, and one-way ANOVA and Dunnett *t*-test (A, B, C) or Bonferroni’s test (D, E) as post hoc tests were applied.

In order to investigate whether SNS could inhibit cortisol-induced breast CSCs and GRP78 expression, 4T1 cells were treated by cortisol combined with gradient concentrations of SNS. It was found that SNS inhibited the stem-like populations in a dose-dependent manner (Figure 5A). Furthermore, in the mammosphere formation assay, SNS attenuated the increase in the number and size of the mammospheres induced by cortisol (Figure 5B). Consistent with the *in vivo* results, SNS inhibited cortisol-activated GRP78 expression in 4T1 cells at both the transcript and protein levels (Figure 5C, Supplementary Figure 3C). Meanwhile, GRP78 overexpression could reduce the anti-CSCs effects of SNS under cortisol stimulation (Figure 5D, Supplementary Figure 3A), as well as the mammosphere formation ability (Figure 5E). Therefore, excessive cortisol induced by CUMS is a key factor contributing to GRP78 overexpression and enhanced breast cancer stemness, and SNS can block the process *via* inhibiting GRP78 expression.

### SNS Interrupts the Interaction Between GRP78 and LRP5 to Suppress Wnt/β-Catenin Signaling

**FIGURE 6:**
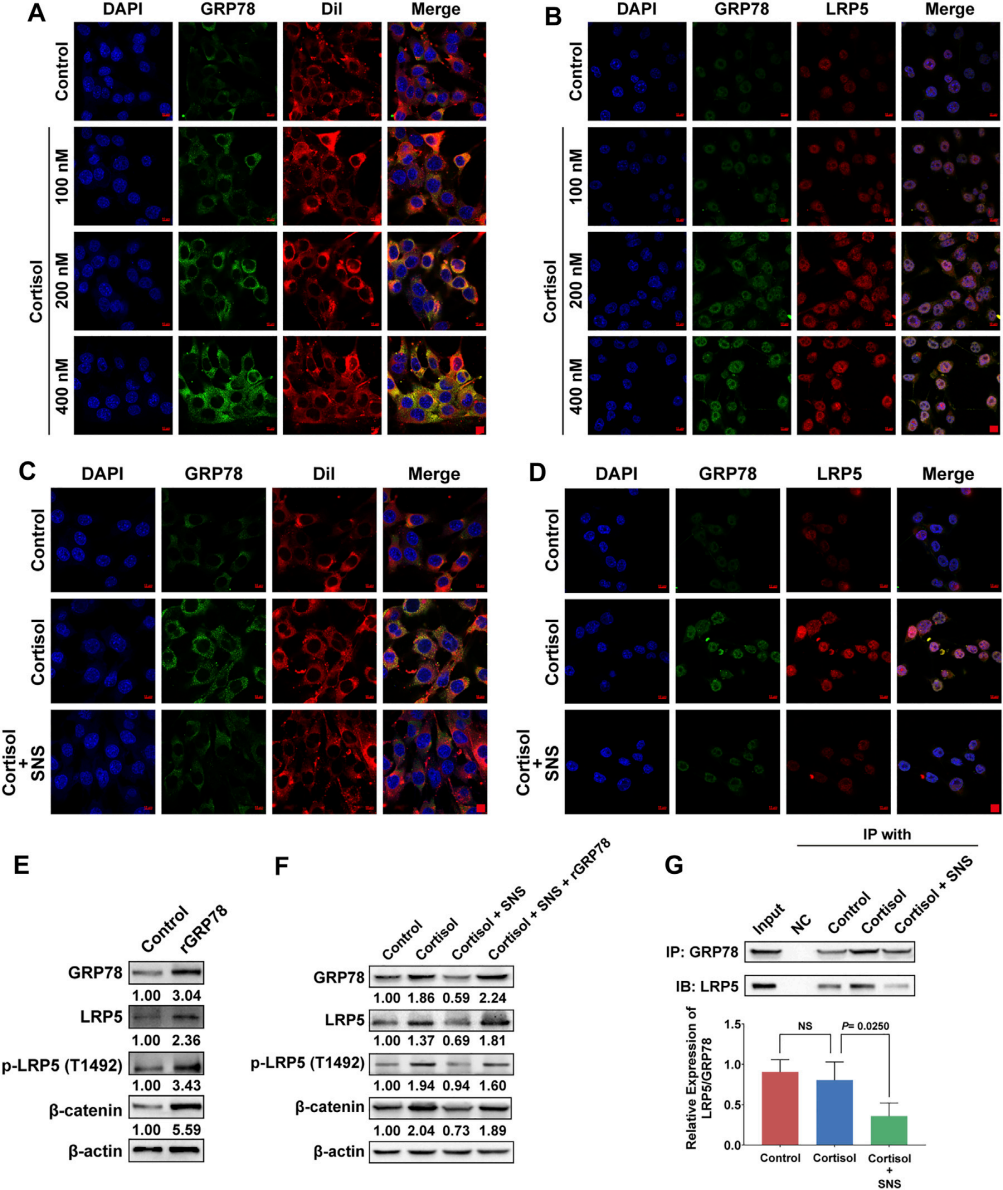
SNS interrupts the interaction between GRP78 and LRP5 to suppress Wnt/β-catenin signaling. **(A)** After 4T1 cells were treated with cortisol for 24 h, the translocation of GRP78 to the cell membrane was monitored by immunofluorescence. Red: DiI-cell membrane tracker; green: GRP78. Co-localization of GRP78 and the cell membrane is shown as yellow fluorescence. **(B)** The 4T1 cells were treated with cortisol for 24 h, and the co-localization of GRP78 and LRP5 was detected by immunofluorescence. Co-localization is shown as yellow fluorescence. **(C)** The effect of SNS (200 μg/ml) on cortisol-induced cell membrane translocation of GRP78 was detected by immunofluorescence following SNS treatment for 24 h. **(D)** The effect of SNS (200 μg/ml) on the co-localization of GRP78 and LRP5 induced by cortisol was detected by immunofluorescence following SNS treatment for 24 h. **(E)** The expression of LRP5, *p*-LRP5, and β-catenin in the GRP78-overexpressing 4T1 cells was measured by western blots. **(F)** The 4T1 cells or GRP78-overexpressing 4T1 cells were treated with SNS (200 μg/ml) for 24 h, and the changes in cortisol-induced LRP5, *p*-LRP5, and β-catenin expression were detected by western blots. **(G)** The 4T1 cells were treated with SNS (200 μg/ml) for 24 h, and changes in the interaction of GRP78 with LRP5 were analyzed by Co-IP assays. Input represents the total protein extracts prepared without the antibody coupling resin. NC indicates the negative control prepared by adding quenching buffer to the antibody coupling resin. NS, not significant. The scale bars indicate 10 μm. One representative experiment of three independent experiments is displayed.

It was reported that GRP78 tends to move towards to cell surface under stress stimulus (Clarke et al., 2019). Our previous study revealed a close relationship between GRP78 and breast CSCs. Therefore, cellular surface GRP78 was considered as a potential receptor for activating CSC signaling. By labeling the cell membrane with DiI, we found that cortisol promoted the expression of cell surface GRP78 in a dose-dependent manner (Figure 6A). More importantly, cortisol enhanced GRP78 and LRP5 signaling expression and promoted their co-localization, as shown by immunofluorescence assays (Figure 6B). However, SNS decreased cortisol-induced GRP78 expression on the cell surface (Figure 6C). In addition, the co-localization of GRP78 and LRP5 promoted by cortisol was attenuated by SNS (Figure 6D). The molecular mechanism study further showed that overexpression of GRP78 promoted the phosphorylation of LRP5 and the expression of downstream β-catenin (Figure 6E, Supplementary Figure 4A). Similarly, cortisol treatment could also activate the LRP5/β-catenin pathway. However, SNS inhibited cortisol-induced Wnt signaling activation. Meanwhile, the inhibitory effect of SNS was rescued by GRP78 overexpression (Figure 6F, Supplementary Figure 4B). Through co-immunoprecipitation assays, it was found that cortisol upregulated the binding level of LRP5 to GRP78, but the ratio of LRP5/GRP78 was not changed significantly. Notably, SNS could inhibit the expression of GRP78, LRP5, and LRP5/GRP78 simultaneously (Figure 6G). In summary, excessive cortisol-induced by chronic psychological stress promoted the expression of GRP78 and its binding to LRP5, subsequently activating CSCs. SNS reduced the expression of GRP78 and interrupted the interaction between GRP78 and LRP5, thus inhibiting the activation of stemness.

### SNS Inhibits CUMS-Mediated Activation of GRP78/LRP5 in Primary and Metastatic Lesions

**FIGURE 7:**
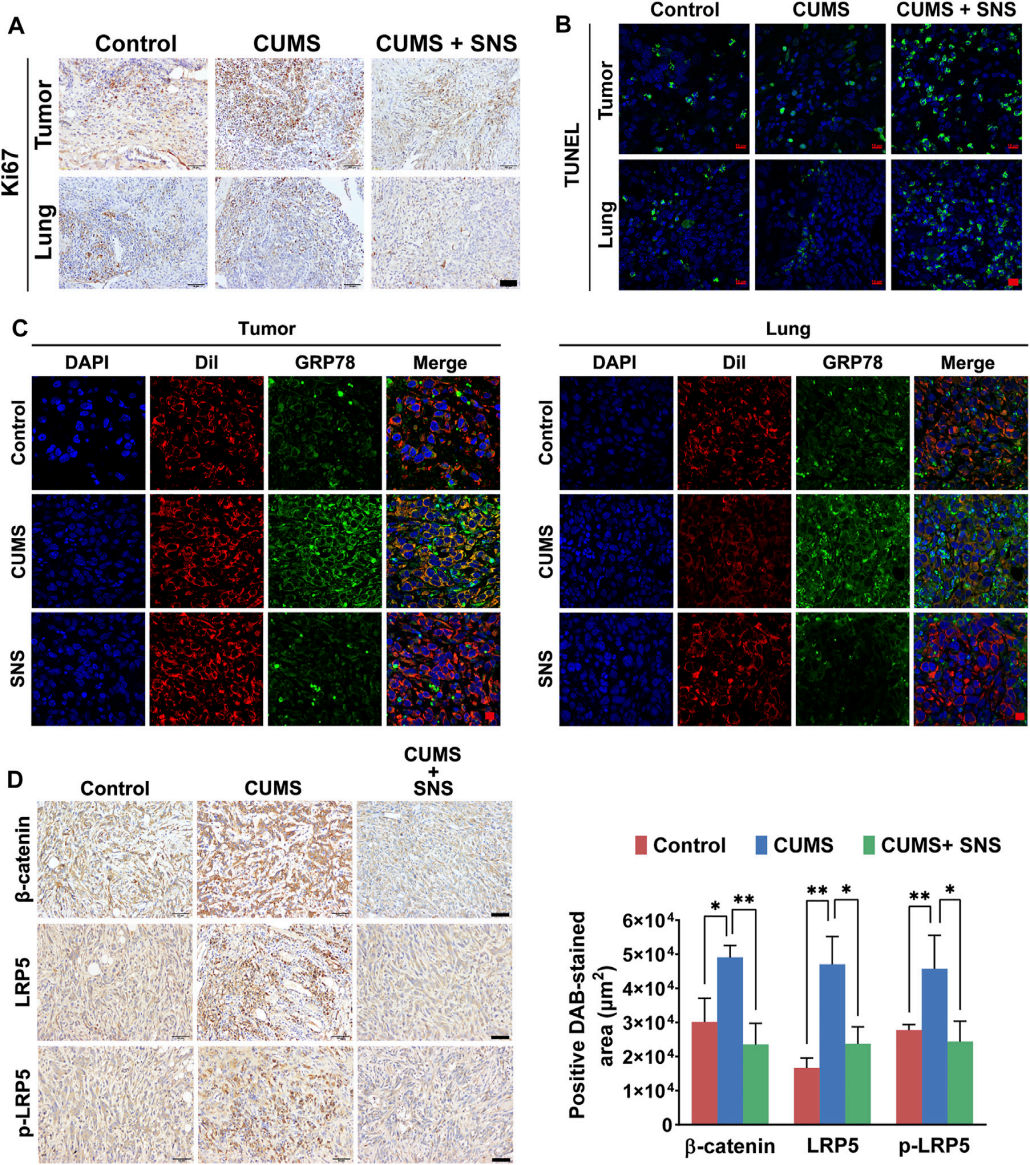
SNS inhibits CUMS-activated Wnt/β-catenin signaling in primary and metastatic lesions of breast cancer in mice. **(A)** The expression of Ki-67 in primary and metastatic lesions of the 4T1 tumor-bearing mice was detected by immunohistochemistry, reflecting the proliferation of breast cancer cells *in vivo* (the scale bars indicate 50 μm). **(B)** The TUNEL assays were used to detect *in situ* apoptosis in primary and metastatic lesions of the 4T1 tumor-bearing mice (the scale bars indicate 10 μm). **(C)** Membrane expression of GRP78 in the primary and metastatic lesions of the 4T1 tumor-bearing mice was detected by immunofluorescence (the scale bars indicate 10 μm). Red: DiI-cell membrane tracker; green: GRP78. Co-localization of GRP78 and the cell membrane is shown as yellow fluorescence. **(D)** The expression levels of LRP5, *p*-LRP5, and β-catenin in the primary tumors were measured by immunohistochemistry (the scale bars indicate 50 μm). Data are represented as the mean value ±SD. One representative experiment of three independent experiments is displayed. One-way ANOVA and Bonferroni’s post hoc test were applied.

The inhibitory effect of SNS on LRP5/β-catenin signaling was needed to confirm *in vivo*. Compared with the non-stressed tumor-bearing mice, the breast cancer cells in the primary lesions and metastatic lesions showed more active cell proliferation under CUMS, as demonstrated by immunohistochemical detection of Ki67. In contrast, SNS suppressed the expression of Ki67 in both the primary and metastatic lesions (Figure 7A). Similarly, SNS enhanced the apoptosis of breast cancer cells in the primary and metastatic lesions, as shown by TUNEL staining (Figure 7B). In addition, through DiI labeling, it was found that CUMS promoted the expression of GRP78 on the cell surface in both primary and metastatic lesions. Similarly, SNS inhibited GRP78 aggregation on the cell membrane induced by CUMS, consistent with the *in vitro* results (Figure 7C). The immunohistochemical results of breast tumors showed that CUMS upregulated the expression of β-catenin and LRP5 and promoted the phosphorylation of LRP5. By contrast, SNS reduced the expression of LRP5 and β-catenin and inhibited the phosphorylation of LRP5, resulting in Wnt/β-catenin pathway inactivation (Figure 7D). Therefore, SNS could inhibit GRP78/LRP5 signaling induced by chronic psychological stress *in vitro* and *in vivo*.

## Discussion

Clinical and epidemiological data of breast cancer in the past 30 years have suggested that chronic psychological stress is a risk factor for the development and metastasis of breast cancer (Cormanique et al., 2015; Joshi et al., 2018). However, there is limited information on how chronic psychological stress affects the metastasis and prognosis of breast cancer. It is generally believed that chronic psychological stress is involved in multiple steps of breast cancer progression, including angiogenesis, invasion, colonization, the immune system, and the tumor microenvironment (Afrisham et al., 2019). The HPA axis and the sympathetic nervous system are the primary systems controlling the psychological stress response and stress-hormone release (Frick et al., 2009; Eng et al., 2014). Catecholamines and glucocorticoids are the main hormones regulated by chronic psychological stress. Adrenergic receptors mainly mediate catecholamines. Therefore, β-blockers have become a potential strategy for tumor therapy, especially for patients with chronic psychological stress. A nested case-control study of 4,113 pancreatic cancer patients and 16,072 matched controls showed that treatment with non-selective β-blockers for more than 2 years reduced the risk of pancreatic cancer (Saad et al., 2020). However, the clinical application of β-blockers is still controversial because of the differences in the effects of different β-blocker subtypes and their therapeutic outcomes on different types of malignancies. The level of glucocorticoids is one of the biochemical hallmarks of chronic psychological stress. Recently, several studies found that glucocorticoids triggered CSCs trait in a GR-dependent manner. GR knockdown was found to block GCs-induced CSCs marker expression and mammosphere formation (He et al., 2019; Kim et al., 2020). However, few studies investigated whether chronic psychological stress-induced glucocorticoid hypersecretion could promote breast cancer stemness. Our current study found that the increased level of cortisol induced by CUMS led to the overexpression of the ER stress marker GRP78, which stimulated the stemness of breast cancer cells to promote metastasis. Therefore, hypersecretion of glucocorticoids is the crucial node linking chronic psychological stress to the CSC signaling network.

Currently, it is believed that breast cancer is essentially a kind of “stem cell” disease with hierarchical heterogeneity driven by CSCs. The self-renewal and production of heterogeneous offspring of breast CSCs are the root causes of breast cancer development and metastasis (Dittmer, 2018; Palomeras et al., 2018). The unique properties and functions of CSCs make them vulnerable to stress, and the stress response is also an important way for CSCs to regulate their self-renewal and differentiation (Kusumoto et al., 2018). It has been suggested that cellular stress activates HIF-1 to promote the expression of DNAJB8, which induces cancer stem cell-like cells (Kusumoto et al., 2018). In addition, oxidative stress, mechanical stress, chronic inflammatory stress, metabolic stress of chronic nutritional exhaustion, and cell replication stress existing in the tumor intracellular environment could lead to ER stress activation in CSCs to maintain their stemness (Tower, 2012). GRP78 is not only a molecular marker of ER stress but also a representative molecular chaperone that can form GRP78/Cripto complex. Studies have shown that Cripto regulates stem cell function by activating the Wnt/β-catenin/Tcf signaling pathway (Rangel et al., 2016; Sandomenico and Ruvo, 2019). GRP78 and Cripto were co-expressed on the head and neck CSCs, and GRP78 knockout was found to attenuate the self-renewal and tumorigenic ability of the head and neck CSCs (Lee et al., 2014). Notably, GRP78 is expressed not only in the cytoplasm but also on the cell membrane when ER stress is activated. Several studies have confirmed that the regulatory effect of Cripto on stem cells depends on its binding to GRP78 on the cell surface. The cell surface localization of GRP78 promoted the stem cell-like activation of cancer cells (Miharada et al., 2011). Moreover, recent studies found that chronic psychological stress also activated ER stress in the brain, liver, and other tissues and upregulated the expression of GRP78 (Mondal et al., 2015; Hosoi et al., 2019). Similarly, our study found that chronic psychological stress elevated the expression of GRP78 and promoted its cell surface localization in mouse breast tumors. The cell surface GRP78 is closely correlated with breast CSCs. Inhibition of cell surface GRP78 reduced the stem cell-like populations and the mammosphere-formation capacity increased by CUMS. Altogether, these results suggested that chronic psychological stress was involved in breast CSCs regulation and that GRP78 was a key factor in stress-induced CSC signaling.

Chronic psychological stress promotes the initiation and progression of breast cancer, which coincides with the cancer pathogenesis theory of TCM. TCM is commonly applied in cancer patients with chronic stress (Cui et al., 2020). In TCM, acupuncture, Tai Chi, and Chinese medicine formulas are the main strategies for relieving chronic psychological stress (Cui et al., 2020). Acupuncture effectively relieved cancer-related discomfort, especially pain in advanced cancer and postoperative pain (He et al., 2020). Tai Chi, as a mindful meditative movement, is similar to psychosomatic behavioral interventions in modern oncology. A randomized, partially blinded, noninferiority trial showed that Tai Chi reduced insomnia and depression in 145 breast cancer patients and was statistically non-inferior to cognitive behavioral therapy for insomnia (Irwin et al., 2017). The application of TCM formulas in cancer patients with chronic psychological stress has also attracted increasing attention. A meta-analysis of 18 randomized controlled trials that included 1441 participants indicated that all 12 tested TCM formulas could alleviate cancer patients’ depressive symptoms to varying degrees (Li et al., 2019). SNS is a classic formula widely applied for relieving depression in TCM. Modern medical studies have shown that SNS combined with fluoxetine more effectively reduced depression-like behaviors in mice than fluoxetine alone, accompanied by the increase of central 5-HT and the decrease of peripheral 5-HT (Li et al., 2013). A meta-analysis of 7 trials suggested that SNS even had a superior outcome than fluoxetine in terms of response rate in patients with poststroke depression (Cai et al., 2021). In addition, a recent study indicated that SNS inhibited increased GCs levels in CUMS model mice by modulating HPA axis (Wei et al., 2016). Although SNS has been applied in TCM clinical practice to reduce the risk of recurrence and metastasis of breast cancer (Ma, 2018; Gao, 2019), the underlying molecular mechanisms remained largely unknown. Our study found that SNS was capable of limiting GRP78 overexpression induced by CUMS, and finally inhibiting breast cancer stemness and metastasis. Of note, it has been reported that chronic psychological stress promoted stem-like properties *via* lactate dehydrogenase A-dependent metabolic rewiring (Cui et al., 2019). Thus, the inhibitory effect of SNS on chronic psychological stress-induced breast cancer stemness might be associated with cellular energy metabolism. It is worth to be explored in our future study. Besides, further investigation is needed to explore the active compounds in SNS targeting GRP78.

**FIGURE 8:**
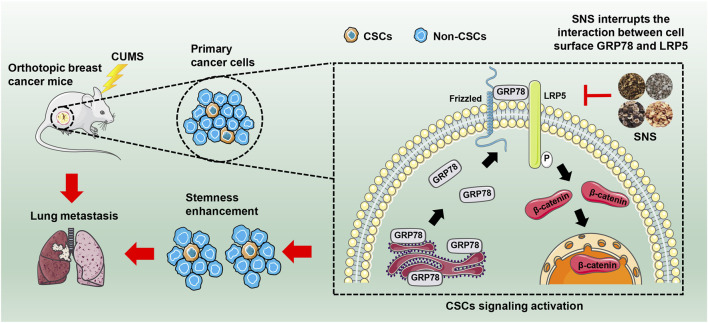
SNS inhibits CUMS-induced lung metastasis and stemness of breast cancer. SNS interrupts the interaction between GRP78 and LRP5 on the cell surface, thus inhibiting the Wnt/β-catenin signaling of breast CSCs.

In conclusion, our study revealed that chronic psychological stress could promote breast cancer stemness *via* activating GRP78-mediated CSCs, and SNS was capable of inhibiting breast cancer stemness and metastasis *via* interrupting the GRP78/LRP5 stem signaling under CUMS stimulation (Figure 8). Our findings not only uncover the biological influence and molecular mechanism of chronic psychological stress on breast CSCs but also highlight SNS as a promising treatment for relieving chronic psychological stress in breast cancer patients to improve their clinical prognosis.

## Data Availability

The raw data supporting the conclusions of this article will be made available by the authors, without undue reservation.
