# Supplementary material for: Sini San Inhibits Chronic Psychological Stress-Induced Breast Cancer Stemness by Suppressing Cortisol-Mediated GRP78 Activation
Source: Front Pharmacol. 2021 Nov 29;12:714163. doi: 10.3389/fphar.2021.714163 (PMC8667778; doi:10.3389/fphar.2021.714163)
Supplement: Supplementary file 1 [file DataSheet1.docx]

Supplementary Material

# Supplementary Figures and Tables

##
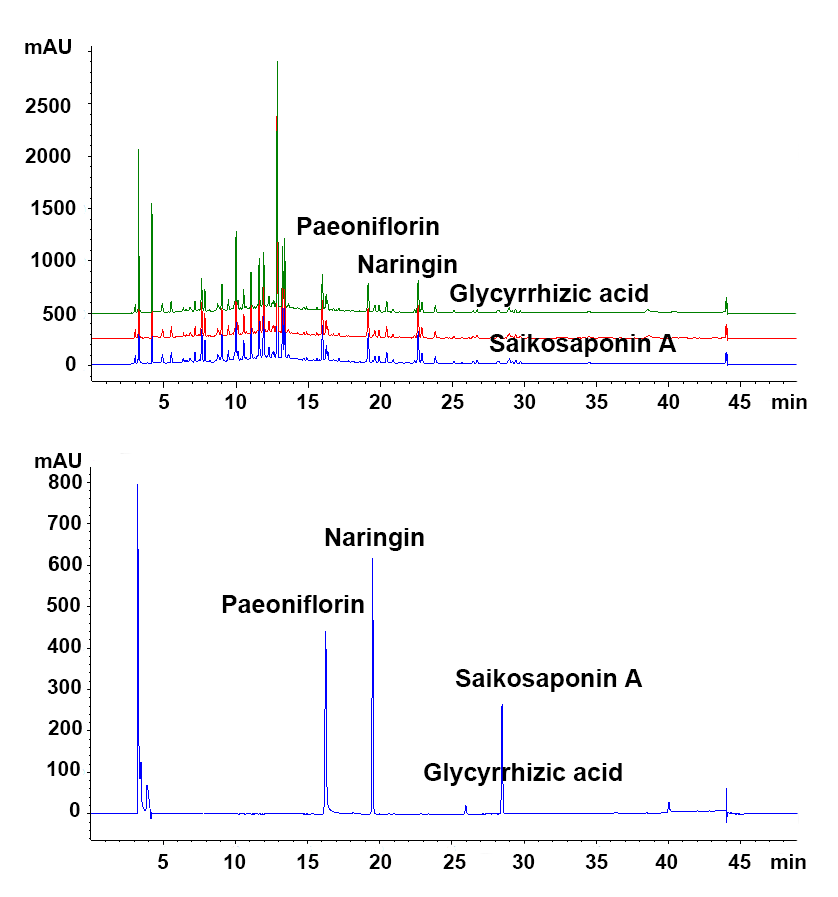
Supplementary Figures

**Supplementary Figure 1.** Fingerprint chromatograms of three independent batches of SNS formula (upper panel) and data for quality control standards (lower panel).


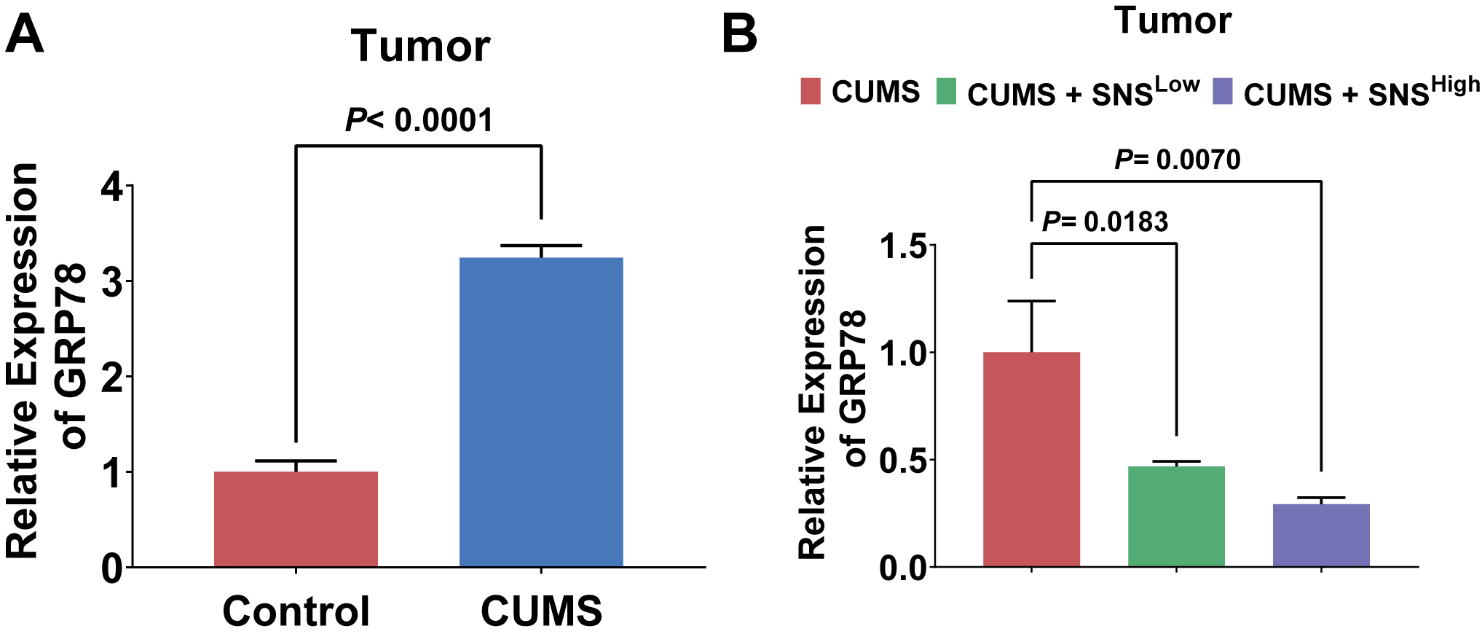


**Supplementary Figure 2.** **(A)** The statistical graphs of the relative protein expression of GRP78 in the primary tumors of the mice under CUMS. **(B)** The statistical graphs of the relative protein expression of GRP78 in the primary tumors of the mice under SNS treatment.


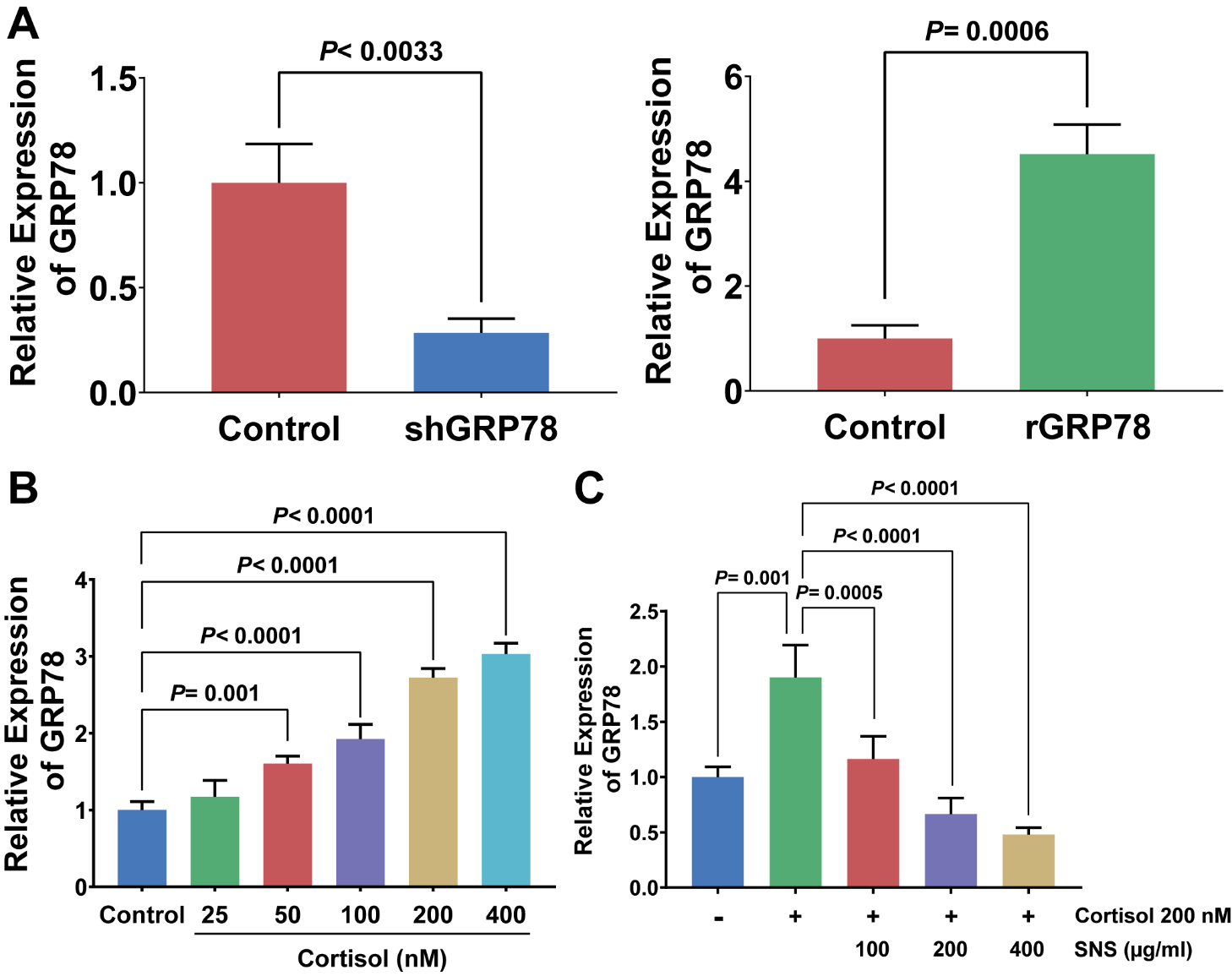


**Supplementary Figure 3.** **(A)** The statistical graphs of the relative protein expression of GRP78 following shRNA knockdown (left panel) or recombinant overexpression (right panel) in 4T1 cells. **(B)** The statistical graphs of the relative protein expression of GRP78 in 4T1 cells treated with cortisol at a gradient concentration. **(C)** Following SNS treatment for 24 h, the statistical graphs of the relative protein expression of GRP78 in the 4T1 cells treated by cortisol were shown.


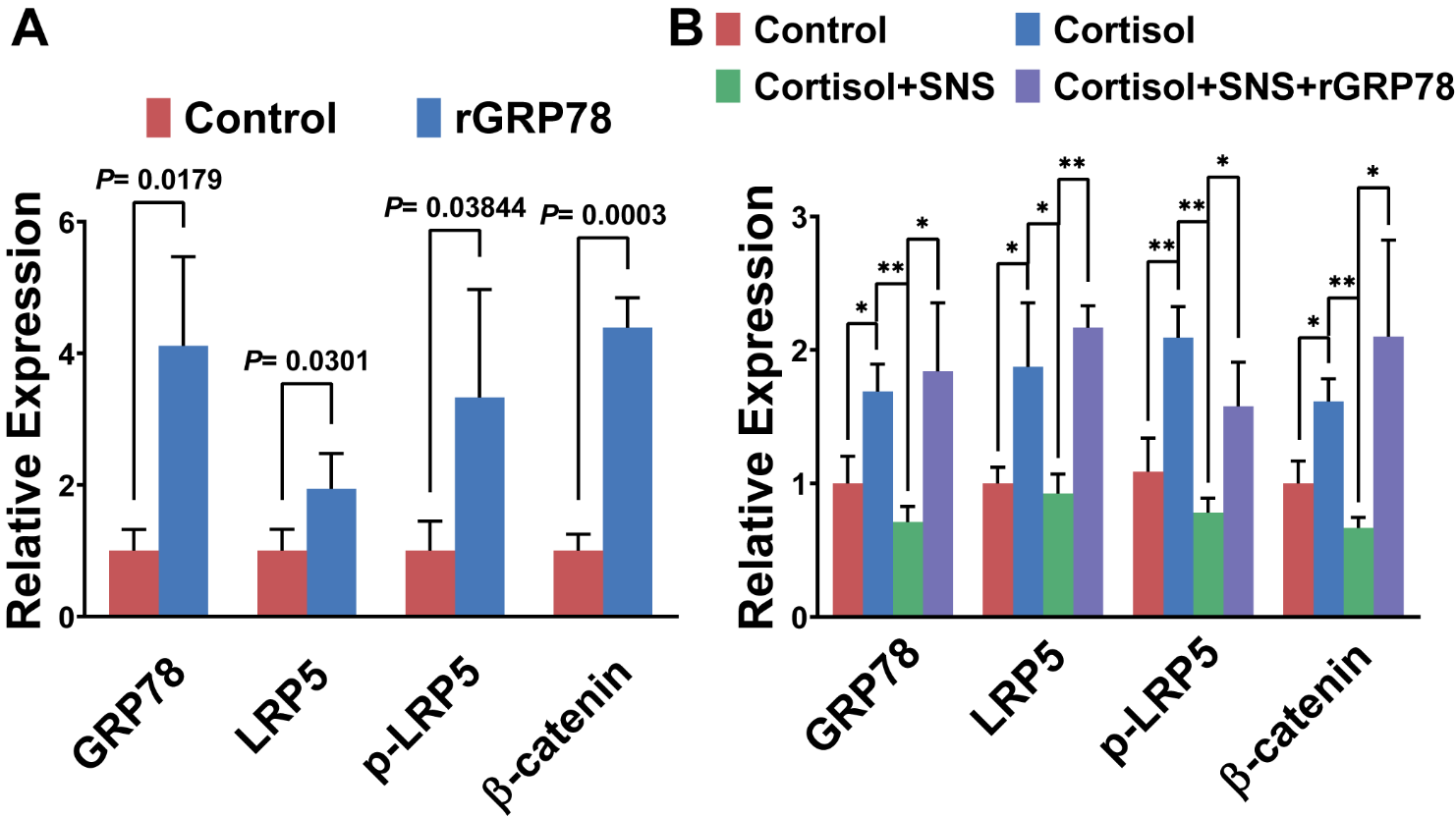


**Supplementary Figure 4.** **(A)** The statistical graphs of the relative protein expression of LRP5, p-LRP5, and β-catenin in the GRP78-overexpressing 4T1 cells. **(B)** The 4T1 cells or GRP78-overexpressing 4T1 cells were treated with SNS for 24 h. The statistical graphs of the relative changes in cortisol-induced LRP5, p-LRP5, and β-catenin expression were shown.

## Supplementary Table

**Table S1. Contents of the standard compounds in three independent batches of SNS formula**

| Analyte | Linear range (ng/mL) | Equation | R^2^ | batch1（g/kg） | batch2（g/kg） | batch3（g/kg） |
| --- | --- | --- | --- | --- | --- | --- |
| Saikosaponin A | 1.95-250 | y = 50214x + 786617 | 0.9937 | 0.0016 | 0.0011 | 0.0007 |
| Naringin | 7.8125-500 | y = 212878x + 1E+07 | 0.9926 | 0.0024 | 0.0031 | 0.0025 |
| Paeoniflorin | 7.8125-500 | y = 32144x + 1E+06 | 0.9951 | 0.0258 | 0.0248 | 0.0221 |
| Glycyrrhizic acid | 7.8125-500 | y = 25749x - 497915 | 0.9925 | 0.0566 | 0.0570 | 0.0551 |
